# Physiological Overview of the Potential Link between the UPS and Ca^2+^ Signaling

**DOI:** 10.3390/antiox11050997

**Published:** 2022-05-19

**Authors:** Dongun Lee, Jeong Hee Hong

**Affiliations:** Department of Health Sciences and Technology, Lee Gil Ya Cancer and Diabetes Institute, Gachon Advanced Institute for Health Sciences & Technology, Gachon University, 155 Getbeolro, Yeonsu-gu, Incheon 21999, Korea; sppotato1@gmail.com

**Keywords:** UPS, calcium, ER stress, ubiquitin, proteasome

## Abstract

The ubiquitin–proteasome system (UPS) is the main proteolytic pathway by which damaged target proteins are degraded after ubiquitination and the recruit of ubiquitinated proteins, thus regulating diverse physiological functions and the maintenance in various tissues and cells. Ca^2+^ signaling is raised by oxidative or ER stress. Although the basic function of the UPS has been extensively elucidated and has been continued to define its mechanism, the precise relationship between the UPS and Ca^2+^ signaling remains unclear. In the present review, we describe the relationship between the UPS and Ca^2+^ signaling, including Ca^2+^-associated proteins, to understand the end point of oxidative stress. The UPS modulates Ca^2+^ signaling via the degradation of Ca^2+^-related proteins, including Ca^2+^ channels and transporters. Conversely, the modulation of UPS is driven by increases in the intracellular Ca^2+^ concentration. The multifaceted relationship between the UPS and Ca^2+^ plays critical roles in different tissue systems. Thus, we highlight the potential crosstalk between the UPS and Ca^2+^ signaling by providing an overview of the UPS in different organ systems and illuminating the relationship between the UPS and autophagy.

## 1. Introduction

The homeostatic maintenance of protein levels or elimination of misfolded or oxidized proteins requires essential quality control processes such as the ubiquitin–proteasome system (UPS) and autophagy [1,2,3,4]. The UPS regulates the intracellular protein levels and mediates the cell cycle modulation, DNA repair, transcription, and apoptosis [5]. Gradationally, the UPS begins with sequential ubiquitination to produce a poly-ubiquitin chain on the target protein [6,7] and is mediated by ubiquitin ligases E1, E2, and E3, which bind ubiquitin to the lysine residues of the target protein [6]. Poly-ubiquitinated proteins are recruited to the 26S proteasome and degraded through proteasome complexes, including the 19S and 20S proteasome [8,9]. The 19S proteasome, which is called the cap of the proteasome, detects poly-ubiquitinated proteins leading to the 20S proteasome, where poly-ubiquitinated proteins are deubiquitinated to recycle ubiquitin proteins [8]. The 20S proteasome, which is composed of α and β subunits, degrades poly-ubiquitinated target proteins [9].

The crosstalk between UPS and oxidative stress has been addressed bilaterally. It is known that inhibitors of UPS induce oxidative or endoplasmic reticulum (ER) stress [10], and protein oxidation through proteasome malfunction has been suggested as a major cause of human diseases such as Alzheimer’s disease (AD) [11], osteoarthritis [12], asthma [13], atherosclerosis [14], and chronic obstructive pulmonary disease [15]. In addition, the activity of UPS is increased by oxidative stress for the degradation of oxidized proteins, and extensive oxidation impairs the components of the UPS [16]. For example, the H_2_O_2_-induced protein carbonyl group, which is the indicator of protein oxidation, is increased by the treatment of proteasome inhibitor MG-132 [17].

The increase of an intracellular Ca^2+^ concentration ([Ca^2+^]_i_) is a messenger signal of oxidative pathways. Oxidative stress triggers an increase of [Ca^2+^]_i_ through the ER membrane Ca^2+^ channels [18], and on the contrary, Ca^2+^ influx induces the generation of reactive oxygen species (ROS) [19]. Ca^2+^ signaling has been extensively studied for the past several decades. Briefly, [Ca^2+^]_i_ is initiated through two pathways via the release of Ca^2+^ from intracellular stores by various extracellular stimuli and the influx of Ca^2+^ through plasma membrane-associated Ca^2+^ channels, including voltage-gated Ca^2+^ channels (VGCCs), ligand-gated Ca^2+^ channels (LGCCs), and Ca^2+^ ATPases [20]. Increased [Ca^2+^]_i_ induces versatile and universal Ca^2+^ signaling to regulate various cellular physiological functions [21], including muscle contraction [22], the release of neurotransmitters [23], T-cell development [24], and fluid secretion [25].

The disruption of proteasome triggers an imbalance of human health and diseases occur [26,27,28,29]. A change of the signaling messenger Ca^2+^ is an essential process in various diseases, including oxidative stress. Thus, we suggest that the studies of basic mechanisms for the UPS with Ca^2+^ establish the foundation for the therapy of proteasome-associated diseases. In the present review, we will discuss the current advances in the roles of Ca^2+^-related proteins and the pathways of Ca^2+^ signaling in the UPS. Although Ca^2+^ signaling and the UPS have critical roles in other organisms, including plants [30,31,32,33] and yeast [34,35,36,37], this review focuses on the mammalian UPS for relating to therapeutic potentials.

## 2. The Relationship between the UPS and Ca^2+^ Signaling

### 2.1. UPS-Mediated Degradation of Ca^2+^-Related Proteins

The UPS and Ca^2+^ signaling are interconnected, since each affects the other. The interconnected nature of these signals plays a critical role in regulating cellular functions. The UPS regulates Ca^2+^ signaling through the degradation of Ca^2+^-related proteins. Ca^2+^ channels and transporters are distributed on the membranes of intracellular organelles or the plasma membrane. In this section, we will discuss the relationship between these systems and how this affects protein degradation.

The endoplasmic reticulum (ER) is a major intracellular Ca^2+^ store. On the ER membrane, inositol 1,4,5-trisphosphate receptor (IP_3_R) releases Ca^2+^ to the cytosol via the binding of released IP_3_ from phosphatidylinositol 4,5-bisphosphate (PIP_2_) [38,39]. Generally, PIP_2_ is hydrolyzed to IP_3_ by phospholipase C (PLC), which is stimulated by the G-protein-coupled receptor, and IP_3_ subsequently activates IP_3_R to release ER Ca^2+^ [38,39]. Ubiquitin ligase ring finger protein 170 (RNF170), which has three membrane-spanning helices, is localized to the ER membrane and binds to IP_3_R [40]. Ubiquitin ligase RNF170-induced UPS downregulates IP_3_R in rat pancreas cells and CHO cells [41,42,43]. A deletion of endogenous RNF170 increased the expression of IP_3_R1 [40]. In other words, the knock-down of RNF170 inhibits IP_3_R ubiquitination and degradation [40]. In addition, reactive oxygen species are involved in the proteasome-associated degradation of IP_3_R. H_2_O_2_ treatment enhances the proteasome-induced degradation of IP_3_R in vascular smooth muscle cells [44]. The treatment of MG-132 recovers the H_2_O_2_-induced degradation of IP_3_Rs [44]. Other ER resident proteins: sarco-/endoplasmic reticulum Ca^2+^-ATPase (SERCAs) and ryanodine receptors (RyRs) are also degraded by the UPS [23,45]. SERCAs are family to the ER-localized P-type cation ATPase that transports cytosolic Ca^2+^ to the ER [46,47]. Inhibition of the UPS with MG-132 increases SERCA expression [23]. Type-2 RyR (RyR2), which contributes to cardiac excitation–contraction coupling, is degraded by the UPS [45]. In the simulated ischemia–reperfusion of mouse cardiomyocytes, RyR2 is degraded by the UPS following the activation of the Ca^2+^-dependent cysteine protease calpain, which is activated during ischemia/reperfusion [45]. Although the studies of the relationship between the UPS and intracellular organelle-releasing Ca^2+^ have been well-developed in the ER, it is meaningful to investigate the effect of the UPS on Ca^2+^ channels and transporters on other intracellular organelles, including the mitochondria and Golgi apparatus.

Regulatory channels of Ca^2+^ signaling, store-operated Ca^2+^ channels (SOCCs), are stimulated by changes in the Ca^2+^ store levels. When the concentration of ER Ca^2+^ is depleted, stromal interaction molecule (STIM) senses the depleted ER, elicits oligomerization, and forms a complex with the Orai channels to induce Ca^2+^ influx [48]. The overexpression of E3 ubiquitin ligase reduces the surface expression of STIM1, and the treatment with MG-132 increases the store-operated Ca^2+^ entry (SOCE), a Ca^2+^ homeostatic process to regulate cellular functions, by rescuing the STIM1 expression [49], whereas the inhibition of the proteasome degrades STIM1 and STIM2 through the complementary activation of autophagy [50]. To maintain the cellular activity by the degradation of proteins, autophagy and the UPS are known to communicate with each other [5]. If one is inhibited, the other is activated to degrade proteins [5]. Thus, the inhibition of the UPS complementally stimulates autophagic flux to maintain the [Ca^2+^]_i_ level. The crosstalk between the UPS and autophagy with Ca^2+^ signaling will be discussed in Section 5.

The N-type Ca^2+^ channel voltage-gated calcium channel (Ca_V_)2.2, which induces peripheral neuron neurotransmission [51], is degraded via the UPS to maintain the precise modulation of its expression [52,53]. For example, the overexpression of Parkin, which is an E3 ligase, decreases the current of Ca_V_2.2 through proteasome-induced degradation [52], and proteasome inhibition through MG-132 increases the current of Ca_V_2.2 [53]. The degradation of Ca_V_2.2 is induced by the light chain of microtubule-associated protein B through ubiquitin-conjugating enzyme E2 L3 (UBE2L3)-mediated ubiquitination [54]. UBE2L3 is an E2-type ubiquitin ligase that is related to the occurrence of various diseases, including rheumatoid arthritis, celiac disease, and Crohn’s disease [55]. The β-subunit of Ca_V_2.2 protects against the excessive degradation of Ca_V_2.2 and even the formation of polyubiquitin chains but not from the binds of one to four ubiquitins [56,57]. Ca_V_1.2 is expressed in the brain, cardiomyocytes, pancreas, adrenal medulla, and bladder smooth muscle [58] and specifically initiates cardiac excitation-contraction coupling [59] and triggers smooth muscle contractions [60]. Similar to Ca_V_2.2, the β-subunit of Ca_V_1.2 promotes the trafficking of Ca_V_1.2 to the plasma membrane to avoid the UPS [61]. The aberrant splicing variant form of the Ca_V_1.2 β-subunit increases the UPS-induced degradation of Ca_V_1.2, which triggers cardiac hypertrophy [62]. Galectin-1 (Gal-1), which reduces the current density of Ca_V_1.2 [63], induces the proteasome-induced degradation of Ca_V_1.2 by disrupting the Ca_V_1.2 β-subunit in HEK 293 cells [64]. Coupling between Ca_V_1.2 and Gal-1 regulates the blood pressure, and Gal-1 deficiency triggers hypertension by activating Ca_V_1.2 in spontaneously hypertensive rats [64]. In conclusion, adjustment of the UPS with the scope of the UPS to regulate Ca^2+^ signaling is proposed as a therapeutic strategy for Ca^2+^ channel-associated diseases, including cardiac hypertrophy and ischemia–reperfusion injury.

### 2.2. Ca^2+^ Signaling and Ca^2+^-Related Proteins Regulate UPS Activity

Ca^2+^ signaling regulates numerous cellular functions. In this section, we will elucidate Ca^2+^ signaling to regulate the UPS. For example, treatment with a Ca^2+^ ionophore (A23187) activates the proteasome within 10 min in ascidian and *Xenopus* eggs [65,66]. Increased proteasome activation is attenuated by the Ca^2+^-chelating agent 1,2-bis(o-aminophenoxy) ethane-N,N,N′,N′-tetra acetic acid (BAPTA)-AM [65,66]. Furthermore, A23187-induced Ca^2+^ increasingly activates the UPS to degrade the signaling proteins, including cyclooxygenase-1 and islet-brain1/JNK interacting protein 1 [67,68]. In neuronal membrane proteasome-inhibited neurons, Ca^2+^ signaling is dominantly attenuated [69]. Similarly, [Ca^2+^]_i_ increases by the constitutive activation of the epithelial sodium channel, which induces the aggregation and activation of caspase-8 to inhibit the proteasome, and activated caspase-8 induces cellular apoptosis [70].

The ER is a major source of increased [Ca^2+^]_i_ that regulates the UPS. Acute ER stress increases the degradation of the amyloid precursor protein, a diagnostic marker of AD [71]. In contrast, human islet amyloid polypeptide aggregation induces ER stress and subsequently impairs the UPS [72]. Aggravated oxidative stress and ER stress produce misfolded proteins in pancreatic β cells and subsequently impair the β-cell function [73]. In summary, the mechanisms by which Ca^2+^-related proteins regulate the UPS can be used to elucidate the interplay between Ca^2+^ signaling and the UPS with the scope of Ca^2+^ signaling to regulate the UPS and may provide dynamic tools for potential therapeutic applications.

#### 2.2.1. Membrane-Bound Proteins and the UPS

Membrane-bound Ca^2+^ channels are categorized into various subfamilies. In this section, we will discuss the membrane-associated Ca^2+^ channels, which regulate the UPS. First, the UPS is regulated by Ca^2+^ signaling from intracellular organelles, including the mitochondria and ER. For example, the treatment with curcumin, which may have anticancer properties [74,75], induces a mitochondrial Ca^2+^ increase, which inhibits the UPS and induces severe vacuolation, which is a marker of paraptosis, along with apoptotic signals, including cellular shrinkage and the generation of apoptotic bodies [76]. Plasma membrane-bound VGCCs are categorized into several subtypes, including L-, N-, P/Q-, R-, and T-type channels [77]. The T-type Ca^2+^ channel inhibitor NNC 55-0396 blocks angiogenesis in human umbilical vein endothelial cells through hypoxia-inducible factor-1 (HIF-1) degradation [78]. Under hypoxic conditions, NNC 55-0396 treatment induces the ubiquitination of HIF-1 and subsequent UPS degradation [78]. Thus, modulation of the T-type Ca^2+^ channels and the subsequent UPS may have therapeutic potential in treating cancer by inhibiting angiogenesis. Transient receptor potential (TRP) channels are nonselective Ca^2+^ channels with various functions and subtypes [79,80] that also regulate the UPS. In oxidative stress induced by ultraviolet irradiation, TRP vanilloid (TRPV)1 is activated and induces an increase in the Ca^2+^ levels in human dermal fibroblasts [81]. The activation of TRPV1 induces the ubiquitination of nuclear factor erythroid 2-related factor 2 (Nrf2), which is a key factor in oxidative stress [81]. In addition, the overexpression of TRPV1 increases the ubiquitination of the epidermal growth factor receptor (EGFR) to reduce EGFR expression [82]. Another plasma membrane channel, the Ca^2+^-sensing receptor (CaSR), which maintains Ca^2+^ homeostasis, also induces proteasome-induced degradation [83]. CaSR inhibits the TGF-beta-dependent phosphorylation of Smad2, which increases its proliferative effect in human embryonic kidney (HEK) 293 cells [83]. The mechanisms by which Ca^2+^ channels and transporters are activated are diverse, and their roles in regulating ubiquitination and the UPS should be studied in further detail. Although the importance of Ca^2+^ channels and transporters is being magnified, the study of the UPS for Ca^2+^ channels and transporters is still attractive.

#### 2.2.2. Cytosolic Ca^2+^-Binding Proteins and the UPS

Cytosolic Ca^2+^-binding proteins involved with the UPS have emerged in various studies. In this section, we will discuss the accumulating evidence of the role of Ca^2+^-binding/related proteins in the modulation of the UPS. The secondary messenger Ca^2+^ delivers signals through Ca^2+^-binding proteins, such as calmodulin (CaM) [84]. CaM is stimulated by the binding of Ca^2+^ and activates Ca^2+^/calmodulin-dependent protein kinases (CaMK) to regulate a variety of physiological functions, including smooth muscle contraction [85], the activation of phosphorylase kinase [86], and activation of the α-amino-3-hydroxy-5-methyl-4-isoxazolepropionic acid receptor [87]. In addition, CaM and CaMK regulate the UPS. E3 ligase, mahogunin ring finger 1 (MGRN1), and glycoprotein 78 (GP78) bind CaM under high [Ca^2+^]_i_, and the treatment with BAPTA attenuates the ubiquitination of MGRN1 and GP78 [88]. CaM bound to MGRN1 and GP78 activates the translocation of GP78 onto the ER membrane to induce ER-associated protein degradation [88]. In hippocampal neurons, the UPS induces an action potential that is inhibited by MG-132 [89]. The treatment with the Ca^2+^/calmodulin-dependent protein kinase II (CaMKII) inhibitor AIPII reduces the rate of protein degradation, while overexpression of the constitutively active form of CaMKII increases the protein degradation [89]. A recent study addressed a new T-type channel enhancer, ethyl-8-methyl-2,4-dioxo-2-(piperidin-1-yl)-2H-spiro[cyclopentane-1,3-imidazo [1,2-a]pyridin]-2-ene-3-carboxylate (SAK3), which has potential therapeutic effects against AD [90]. CaMKII is a scaffold protein that phosphorylates the proteasome subunit Rpt6 [91]. The administration of SAK3 increases CaMKII-Rpt6 signaling, which enhances the proteasome activity in dendritic cells [90]. In myotubules, the Ca^2+^ ionophore A23187 induces the UPS, while CaMKII inhibitors KN-62 and KN-93 dominantly attenuate the proteasome activity [92].

During muscle wasting caused by cachexia, the Ca^2+^-binding protein calpain induces Ca^2+^-dependent proteolysis and the breakdown of myofibrillar proteins [93]. The calpains activate ER-bound transcription factor 11 (TCF11)/Nrf1, which activates the 26S proteasome subunit genes [94]. Calpain-1 cleaves TCF11/Nrf1 to generate the active form, and the inhibition of calpain-1 slows down the degradation of TCF11/Nrf1 [94]. Another ER-related protein RNF122 interacts with Ca^2+^ to modulate the cyclophilin ligand (CAML) to stabilize RNF122, thus inhibiting the ubiquitination of RNF122 [91]. The lectin chaperone calreticulin, which maintains [Ca^2+^]_i_ homeostasis, regulates the proteasome activity [95]. In calreticulin-deficient cells, the number of ubiquitinated proteins and proteasome activity are increased [95]. In addition, the Ca^2+^-binding protein S100, which regulates the tumor cell viability [96], interacts with the E3 ubiquitin ligase C-terminus of the Hsc70-interacting protein (CHIP) to inhibit ubiquitination and the proteasome system [97]. The current understanding of Ca^2+^ signaling and its associated proteins in the UPS is summarized in Table 1.

## 3. Ubiquitination in Organ Systems

The UPS is expressed in various mammalian tissues, including the pituitary gland [98], liver [99,100], lung [101], kidney [101], skeletal muscle [102], lens [103], and placenta [104]. In this section, we summarize the physiological roles of the UPS with Ca^2+^-mediated proteins in various organ systems based on experimental evidence.

### 3.1. The Nervous System

The UPS regulates the nervous system through the modulation of nerve cell activity [105]. The inhibition of voltage-gated sodium channels or gamma-aminobutyric acid (GABA) receptors induces the UPS-mediated degradation of postsynaptic density proteins in rat hippocampal neurons [106]. Keil et al. demonstrated that STIM1 is expressed in hippocampal neurons and is a candidate for synaptic ubiquitinated proteins [49]. Measurement of the Ca^2+^ influx through SOCE in the presence of MG-132 proteasome inhibitors shows increases of the surface STIM1 [49]. The proteasome localizes to the neuronal plasma membrane to induce neuronal Ca^2+^ signaling [69]. In addition, inhibition of the UPS induces autophagy, subsequently leading to the degradation of STIM1/STIM2, which causes neurodegenerative diseases, including AD and Parkinson’s disease [50]. Peptides that are generated by the degradation of intracellular proteins via the involvement of proteasomes are delivered into the extracellular matrix and, subsequently, stimulate neuronal signaling through Ca^2+^ increases [69]. Mutations of the ER membrane-associated ubiquitin ligase RNF170 cause neurodegeneration through the inhibition of IP_3_-induced Ca^2+^ signaling [107]. Neuronal activity is also regulated by CaM [89,90,91,108]. The constitutively active CaMKII increases the proteasome activity by the phosphorylation of the proteasome and recruits the proteasome to hippocampal neurons [89,91]. Moreover, CaMKII activation induces proteasome activation to improve spinal abnormalities [90]. Interestingly, in the case of Ca_V_2.2, the voltage-gated Ca^2+^ channel β-subunits of Ca_V_2.2 protect Ca_V_2.2 from proteasome-induced degradation in sympathetic neurons [57]. The activation of UPS is related to the positive or negative regulation of Ca^2+^ signaling, according to the location of neuronal tissues based on the current evidence. Thus, the verification of UPS-related Ca^2+^ signaling in the nervous system is still a challenging issue, and expanding our understanding of the UPS may contribute towards the more effective treatments of neuronal diseases.

### 3.2. Muscle and Cardiovascular Systems

The UPS regulates muscle atrophy through the degradation of myofibrillar proteins to mediate myogenesis [109]. In addition, the UPS induces the loss of skeletal muscle mass [110] and myocardial remodeling [111]. UPS inhibition causes cardiac dysfunction and heart failure [112]. Since Ca^2+^ is the main driver of muscle contraction [113], various Ca^2+^-dependent proteins regulate muscular and vascular functions through the involvement of UPS. Inhibition of the UPS with MG-132 in rat cardiac cells increases the levels of the Ca^2+^ channels and transporters, including SERCA2, Na^+^/Ca^2+^ exchanger (NCX)1, and RyR2 [114]. A UPS malfunction, through mitochondrial stress, oxidative stress, cytotoxic reagents, or infection, causes cardiac dysfunction through protein aggregation, electrophysiological dysfunction, and the accumulation of cardiac remodeling proteins [112]. Activation of the UPS with dexamethasone, which impairs post-injury skeletal muscle regeneration, occurs through an increase in calpain in the myotubes [115]. In addition, a simple increase in [Ca^2+^]_i_ through the A233187 Ca^2+^ ionophore increases the proteasome activity via CaMKII- and calpain-dependent mechanisms [92]. SERCA, which is a main component protein of the skeletal muscle, maintains Ca^2+^ homeostasis in the skeletal muscle [116]. SERCA malfunctions caused by a missense mutation in the ATP2A1 gene, which encodes SERCA isoform 1, induce Chianina cattle congenital pseudomyotonia muscular disorder, which impairs muscle relaxation [117], whereas the inhibition of the UPS with MG-132 results in an increased expression of SERCA1 and increased activity of Ca^2+^-ATPase [23]. The treatment with MG-132 attenuates carbachol (which induces Ca^2+^ release from sarcoplasmic reticulum (SR) [118])-induced Ca^2+^ signaling in SERCA1 mutant-transfected cells, indicating that MG-132 recovers the SERCA1 expression to restore the ER Ca^2+^ concentration from the cytoplasm [23]. IP_3_R activates calmodulin to phosphorylate the myosin light chain during muscle contractions [119]. Decreased IP_3_R expression via H_2_O_2_ attenuates the vascular reactivity in rat thoracic aortic rings [44]. In addition, the levels of SR protein RyR2, which regulates excitation–contraction coupling and cardiac cell recovery [120,121], decrease in the heart after ischemia/reperfusion [122,123]. Pedrozo et al. demonstrated that the activation of the UPS through calpain causes the degradation of RyR2 proteins in SR [45]. In addition, Ca_V_1.2 induces Ca^2+^ influx to stimulate smooth muscle contractions [60] and regulate the arterial blood pressure [124]. Ca_V_1.2 is inhibited by Gal-1, which displaces the β-subunits of Ca_V_1.2, disturbing its protective role against the UPS [64], and its deficiency triggers high blood pressure [125]. Gal-1, therefore, modulates the expression of Ca_V_1.2 through the UPS to maintain the blood pressure [64]. Considering Ca^2+^ is a major resource for muscle contraction, UPS should be an important process to sustain Ca^2+^ homeostasis in muscles and cardiovascular tissues. Thus, a wide range of Ca^2+^-related proteins and Ca^2+^ signaling interact with UPS in the muscle, and the cardiovascular system provides direct evidence of the interaction between the UPS and Ca^2+^ signaling and, thus, should be extensively elucidated for future study.

### 3.3. The Pancreas

The pancreas is one of the exocrine glands and has a role in digestion and glucose metabolism. Although UPS-related experimental evidence is relatively unknown in pancreatic glands, activation of the UPS downregulates IP_3_R in rat pancreatic islet cells [42]. During the development of pancreatic cells, pancreatic and duodenal homeobox 1 (Pdx1) is a marker of pancreatic progenitor cells [126] and is necessary for β-cell maturation [127]. The proteasome-induced degradation of Pdx1 is protected by the involvement of Ca^2+^ sensor secretagogin in β cells [128]. Increases in [Ca^2+^]_i_ in β cells induce the apoptotic pathway through the UPS [68,72]. When [Ca^2+^]_i_ increases through Ca^2+^ ionophore A23187, or by the supplementation of Ca^2+^ in the media in pancreatic β cells, the UPS-mediated degradation of islet-brain 1 and c-Jun N-terminal kinase (JNK) interacting protein 1 (IB1/JIP1) is increased [68]. The IB1/JIP1 are antiapoptotic scaffold proteins and block the JNK pathway [129]. Mutations of IB1/JIP1 induce the activation of the JNK pathway to trigger the apoptosis of β cells and subsequently induce type 2 diabetes [129]. In addition, the increase in [Ca^2+^]_i_ that occurs through ER stress reduces the proteasome activity in β cells [72]. Impairment of the proteasome activity induces the aggregation of the extracellular human islet amyloid polypeptide (hIAPP), which finally induces β-cell apoptosis [72], suggesting that pancreatic β-cell homeostasis is closely related to Ca^2+^ signaling and proteasome activity. More recently, nicardipine, a drug to treat high blood pressure that is also known as an L-type Ca^2+^ channel blocker, blocked the proteasome through CaMKII and increased [Ca^2+^]_i_ in pancreatic acinar cells [130], suggesting that the treatment of nicardipine should be considered in unwanted pancreatic acinar cell damage. Thus, although direct evidence is rare, verification of the precise mechanism between the UPS and Ca^2+^ signaling in the pancreas might provide potential strategies for diabetic treatment and pancreatic injury.

### 3.4. Other Tissues

Several tissues, including reproductive cells, osteoblasts, and mesangial cells, are affected by the relationship between the UPS and Ca^2+^ signaling. In the meiotic cell cycle, [Ca^2+^]_i_ affects proteasome activity [65]. Briefly, the treatment with A23187 in metaphase-anaphase transition transiently induces the modulation of proteasome activity, and BAPTA-AM co-treatment sustains the proteasome activity [65].

Inhibition of the UPS by MG-132 blocks the forskolin-mediated decrease of core binding factor α-1 (Cbfa1) [131]. Briefly, core binding factor α-1 (Cbfa1) is a master regulator of osteoblastic differentiation [132,133]. Cbfa1 expression is decreased in forskolin-treated osteoblastic cell lines [131]. The treatment with forskolin increases cyclic adenosine monophosphate (cAMP), which is stimulated by [Ca^2+^]_i_ and induces Ca^2+^ signaling [134]. Ca^2+^-related cAMP thus regulates Cbfa1 expression through the UPS to inhibit osteoblastic differentiation [131].

In addition to the renal system, the treatment of mesangial cells with high glucose attenuates Orai1 expression through ubiquitination of Orai1, whereas MG-132 recovers the expression of Orai1 [135]. A high glucose treatment induces dysregulated SOCE of the mesangial cell through the UPS-mediated degradation of Orai1 [135]. The maintenance of SOCE in the renal system is a critical process against diabetic injury. Above all, although the interaction between the UPS and Ca^2+^ signaling is multifaceted, their precise modulation according to each specific tissue type requires additional research.

## 4. Ubiquitination in Cancer

The UPS, which degrades tumor suppressor proteins, contributes to the development and sustaining of the cancerous phenotype [136,137], whereas inhibition of the UPS through the knockdown of UPS-related proteins decreases cancer cell survival [138,139]. Thus, proteasome inhibition has been suggested as a potential target for cancer therapy [140,141]. In this section, we will discuss the relationship between the proteasome and Ca^2+^ signaling in cancer cells based on the experimental evidence. In triple-negative breast cancer cells, inhibition of the proteasome induces cancer cell death via the involvement of several Ca^2+^-signaling pathways. The proteasome inhibitor bortezomib (BTZ) induces 5′-adenosine monophosphate (AMP)-activated protein kinase (AMPK) activation by increasing [Ca^2+^]_i_ and CaMKII subunit β in MDA-MB-231 and MDA-MB-498 cells [142]. Indirubin-3-monoxime (I3M, which enhances apoptosis through oxidative stress [143]) and curcumin are known anticancer components that induce cancer cell apoptosis [143,144]. These compounds induce paraptosis through mitochondrial Ca^2+^ overload accompanied by proteasome impairment in malignant breast cancer cells [76,145]. Paraptosis is a type of programmed cell death that initiates cytoplasmic vacuolization, which is generated from the ER and mitochondria [76]. I3M induces proteasome impairment-mediated ER stress and the transference of ER Ca^2+^ into the mitochondria through a mitochondrial Ca^2+^ uniporter, which causes paraptosis in MDA-MB-231 cells [145]. Similarly, curcumin inhibits proteasome to increase the mitochondrial Ca^2+^ overload, which is followed by paraptosis in MDA-MB-435S and MDA-MB-231 cells [76]. In human liver carcinoma cells, NNC 55-0396, a T-type Ca^2+^ channel blocker, inhibits tumor angiogenesis through the UPS-induced degradation of HIF-1 [78].

## 5. The Crosstalk between the UPS and Autophagy with Ca^2+^ Signaling

To understand the relationship between the UPS and Ca^2+^ signaling in the cellular clearance system, autophagy as another clearance system should be also illuminated. Thus, in this section, we describe the crosstalk between the UPS and autophagy in Ca^2+^ signaling. Several researchers have proposed that the UPS is associated with the autophagic process [5,146,147]. In mammalian cells, there are three types of autophagy, including macroautophagy, chaperone-mediated autophagy, and microautophagy [148]. Although three types of autophagy are distinct in mechanisms, all types of autophagic mechanisms are based on lysosomal protein degradation and recycling [149]. The relationship between the UPS and autophagy has to be further clarified to understand the details of interconnection between the UPS and autophagy. Thus, in this review, we deal with only macroautophagy, which is extensively studied. Macroautophagy proceeds via two components, the autophagosome and the lysosome, which are stimulated by starvation and the mTORC1 complex [150]. The autophagosome recruits target molecules to bind p62 [151,152,153], and the lysosome contains various hydrolases to degrade proteins. Autophagosome and lysosome fusion occurs during autophagy and performs a degradative function [154,155].

Similar to the UPS, Ca^2+^ signaling is the key signaling modulator of autophagic flux through lysosomal Ca^2+^ release, which is a critical cellular component of autophagy [156,157,158]. The lysosome is a small cellular compartment that sustains the luminal pH and contains several ions that are essential for lysosomal activity [159]. Most importantly, lysosomal Ca^2+^ channels play a pivotal role in various cellular physiological functions, as well as lysosomal functions [160]. The regulatory role of Ca^2+^ in autophagy has been extensively studied [160,161,162,163,164]; however, the relationship between the UPS and lysosomal Ca^2+^ signaling is rarely studied. The crosstalk of the UPS and autophagy occurs to sustain the protein activity and to supplement proteolysis where needed [5,50]. In particular, p62, which is a major component of autophagy, is associated with the UPS [147]. When the UPS is downregulated, the p62 activation is increased and then competes with Nrf2 for Kelch-like ECH-associated protein 1 (Keap1) [147]. The p62–Keap1 complex triggers the aggregation of ubiquitinated proteins in the UPS [147]. The overexpression of p62 enhances the activity of the UPS to form aggregates of ubiquitinated proteins, whereas the downregulation of p62 inhibits the UPS [147]. Crosstalk between the UPS and autophagy via Ca^2+^ signaling is, therefore, an essential interaction for maintaining cellular homeostasis.

**Figure 1:**
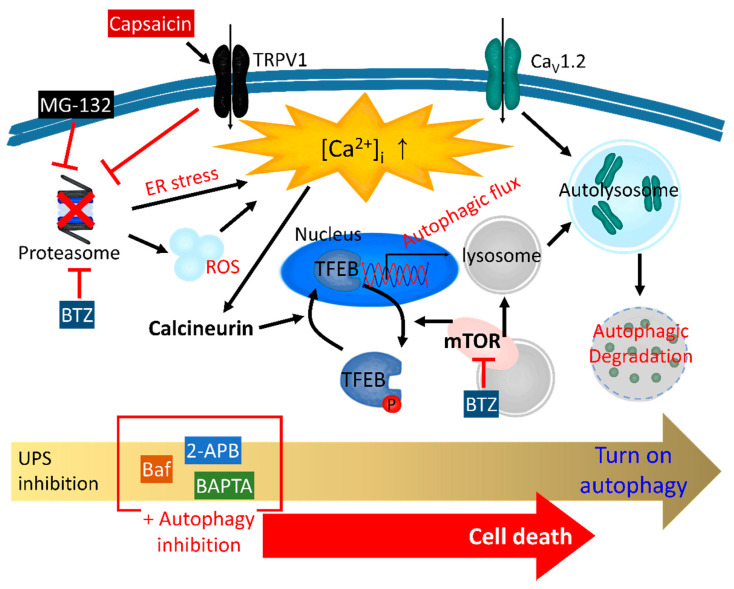
The crosstalk between the UPS and autophagy in the presence of Ca^2+^ signaling. The schematic illustration demonstrates the relationship between the UPS and autophagy through proteasome inhibitors in the presence of oxidative or ER stress and the subsequent intracellular Ca^2+^ increase. The treatment of MG-132, BTZ, and TRPV1 agonist capsaicin inhibits the proteasome activity. The inhibition of the proteasome induces ER stress, which, finally, stimulates calcineurin activity. Calcineurin triggers the dephosphorylation of TFEB to induce the translation associated with autophagy, and BTZ blocks the inhibitory effect of mTOR, which maintains the phosphorylation of TFEB. The inhibition of the proteasome is accompanied by an autophagic flux, which, finally, degrades Ca_V_1.2. Thus, the simultaneous inhibition of the proteasome and autophagy destroys the protein homeostasis to induce cell death. 2-APB, 2-aminoethyldiphenylborinate; Baf, bafilomycin A1; BAPTA, 1,2-bis(o-aminophenoxy) ethane-N,N,N′,N′-tetra acetic acid; BTZ, bortezomib; Ca_V_1.2, voltage-gated calcium channel 1.2; TFEB, transcription factor EB; TRPV1, transient receptor potential vanilloid 1; ROS, reactive oxygen species.

The ER stress-related regulation of proteolysis has been thoroughly investigated. Treatment with the UPS inhibitor MG-132 increases [Ca^2+^]_i_ via ER stress in HCT116 colon cancer cells [165] and generates ROS in C6 glioma cells [166] (Figure 1). MG-132 induces cellular vacuolization, leading to autophagosome–lysosome fusion, which is impaired by Ca^2+^ chelation through BAPTA-AM [165]. Additionally, in hormone receptor-positive breast cancer MCF-7 cells, MG-132 induces autophagy through ER stress and, subsequently, apoptosis [167]. The accumulation of misfolded proteins and perturbed unfolded protein response in the ER causes ER stress and produces ROS, and these misfolded proteins are generally degraded by UPS or autophagy [168]. The downregulation of one side of the pathway induces the supplementary upregulation of the other side, so that, when the UPS is inhibited, the autophagy is increased. Consequently, the inhibition of both the UPS and autophagy induces cell death. A combination of UPS inhibitors, including BTZ, and autophagy inhibitors, including bafilomycin A1 (Baf) or 2-aminoethyldiphenylborinate (2-APB), may therefore be an effective clinical cancer therapeutic strategy [169,170]. The treatment with only Baf (10 nM, 48 h) and only BTZ (10 nM, 48 h) decreases the cellular viability by approximately 20% in U266 myeloma cells [169]. However, a co-treatment with Baf (10 nM) and BTZ (10 nM) for 48 h results in a remarkable decrease in the viability of approximately 90% [169]. The combination of BTZ and 2-APB results in enhanced cell death compared to the treatment with BTZ alone in A549 lung cancer cells [170] (Figure 1). In addition, a co-treatment with BTZ and 2-APB decreases the lung tumor volume and weight in vivo compared to BTZ or 2-APB treatment alone [170]. Malfunction of the proteasome through BTZ increases the Ca^2+^-related protein activity, including calcineurin, and activates autophagy through the calcineurin-transcription factor EB-p62 pathway in cardiomyocytes (Figure 1) [171,172]. In a recent study, ubiquitinated Ca_V_1.2 was degraded by autophagy through the ubiquitin-binding proteins RFP2 and p62 (Figure 1) [173]. p62 senses ubiquitinated proteins in order to degrade Ca_V_1.2 through autophagy and induces the action potential duration [173]. In thymus cells, the stimulation of TRPV1 via capsaicin reduces the proteasome activity (Figure 1) [174]. However, the induction of autophagy reverts capsaicin-induced UPS inhibition [174]. In addition, the deletion of TRPV1 attenuates both the UPS- and autophagy-related protein levels [174]. In this case, the UPS and autophagy do not contribute to the complementary proteolysis, and TRPV1 obviously modulates both the UPS and autophagy. Thus, the lineage of UPS-autophagy-Ca^2+^ signaling reveals a convergence and may also be required to overcome its complexity.

## 6. Conclusions

The studies of the relationship between the UPS and Ca^2+^ signaling propose the key mechanism to maintain cellular homeostasis as a cellular clearance system. It is well-known that oxidative stress and Ca^2+^ signaling have a mutual interplay, including increases of [Ca^2+^]_i_ from the ER and the stimulation of mitochondrial ROS [18,175]. From the point of view that oxidative stress interacts with the UPS, an understanding of the UPS and Ca^2+^ signaling is needed to comprehend the delicate signaling modulation. Various organ systems have been elucidated with regards to UPS regulation. Briefly, in the nervous system, protein aggregation is considered the hallmark of neurodegeneration, and the key proteins, which are associated with Huntington’s disease (mutant huntingtin), Parkinson’s disease (α-synuclein), and amyotrophic lateral sclerosis (superoxide dismutase), are substrates of the UPS [176]. Additionally, inhibition of the UPS induces inflammatory toxicity (lymphopenia [177]), cardiomyopathies (arrhythmia [178]), the depletion of alloreactive T cells [179], and ischemia–reperfusion injury [180]. Although protein aggregation could be attenuated by activation of the UPS, paradoxically, proteasome inhibitor BTZ has been suggested as a potential drug for cancer therapy [181]. A recent study suggested that proteasome inhibitors could be therapeutic targets for various diseases, including infectious diseases, autoimmune diseases, and neurodegenerative diseases [182]. However, the clinical approaches of proteasome inhibitors should be carefully considered due to their limitations [182]. Especially, BTZ treatment occurs alongside the damage of nerves, including peripheral neuropathy [183,184], and causes a dose limitation of BTZ when treating myeloma patients [185]. Thus, we suggest that the verification of Ca^2+^ signaling as the checklist of clinical approaches in proteasome modulation might be beneficial to avoid unwanted effects.

## Figures and Tables

**Table 1:** The effect of Ca^2+^ signaling on the UPS.

Related Signaling	Effect on UPS	Details	Ref
Mitochondrial Ca^2+^ release	Inhibition	Curcumin inhibits the UPS to induce paraptosis.	[76]
T-type Ca^2+^ channel	Inhibition	NNC 55-0396 inhibits T-type Ca^2+^ channels to attenuate cancer angiogenesis.	[78]
TRPV1	Activation	Activation of TRPV1 induces the ubiquitination of Nrf2.	[81]
		Overexpressed TRPV1 increases the ubiquitination of EGFR to induce the UPS.	[82]
CaSR	Activation	CaSR maintains Ca^2+^ homeostasis through the UPS.	[83]
CaM	Activation	CaM induces the translocation of GP78 for ER-associated UPS.	[88]
CaMKII	Activation	Phosphorylation of Rpt6 through CaMKII enhances the UPS.	[90]
Calpain	Activation	Calpain-induced activation of Nrf1 stimulates the 26S proteasome subunit gene.	[94]
CAML	Inhibition	CAML stabilizes RNF122.	[91]
Calreticulin	Inhibition	Deficiency of calreticulin increases the UPS.	[95]
S100	Inhibition	Inhibition of the E3 ubiquitin ligase.	[97]

Abbreviations: TRPV1, transient receptor potential vanilloid 1; CaSR, Ca^2+^-sensing receptor; CaM, calmodulin; CaMKII, Ca^2+^/calmodulin-dependent protein kinase II; CAML, Ca^2+^-modulating cyclophilin ligand; UPS, ubiquitin proteasome system; Nrf, nuclear factor erythroid 2-related factor; GP78, glycoprotein 78; RNF122, ring finger protein 122.

## Data Availability

Not applicable.
